# Projected US Urology Workforce per Capita, 2020-2060

**DOI:** 10.1001/jamanetworkopen.2021.33864

**Published:** 2021-11-16

**Authors:** Catherine S. Nam, Stephanie Daignault-Newton, Kate H. Kraft, Lindsey A. Herrel

**Affiliations:** 1 Department of Urology, Michigan Medicine, University of Michigan, Ann Arbor

## Abstract

**Importance:**

Projections to 2035 have demonstrated concern regarding a worsening urology workforce shortage.

**Objective:**

To project the size and demographic characteristics of the urology workforce per capita into 2060 and to anticipate the timing and degree of the impending urology workforce shortage.

**Design, Setting, and Participants:**

This population-based cross-sectional study used the 2019 American Urological Association Annual Census data and the Accreditation Council for Graduate Medical Education’s Data Resource Book from 2007 to 2018. The cohort included practicing urologists in 2019. US Census data were used to approximate the projected US population. Data analysis was performed from June 2020 to March 2021.

**Exposures:**

Continued growth stock and flow model of 13.8% and stagnant growth model of 0% increase of the incoming urology workforce with cohort projection per projected US population.

**Main Outcomes and Measures:**

The primary outcome was urology workforce projection per the population aged 65 years and older. Urology workforce projections per capita and demographic characteristics of the urology workforce up to 2060 were calculated under guided assumptions with 2 stock and flow models.

**Results:**

In 2019, there were 13 044 urologists (11 758 men [90.1.%]; 1286 women [9.9%]; median age range, 55-59 years), with 3.99 urologists per 100 000 persons and 311 new urologists entering the workforce. In a continued growth model, 2030 will have the lowest number of urologists per capita of 3.3 urologists per 100 000 persons, and recovery to baseline will occur by 2050. There are 23.8 urologists per 100 000 persons aged 65 years and older in 2020, which decreases to 15.8 urologists per 100 000 persons aged 65 years and older in 2035 and never recovers to its baseline level by 2060. In a stagnant growth model, there will be a continued decrease of urologists per capita to 3.1 urologists per 100 000 persons by 2060. There is a continued decrease in per capita urologists at each time point, with 13.1 urologists per 100 000 persons aged 65 years and older by 2060.

**Conclusions and Relevance:**

With the impending urology workforce shortage, there will be an exaggerated shortage of total urologists per persons aged 65 years and older in both models. This projection highlights the need for structural changes and advocacy to maximize the available urology workforce.

## Key Points

**Question:**

What are the projected size and demographic characteristics of the urology workforce per capita in the US through 2060?

**Findings:**

In this cross-sectional study, 2 stock and flow models of continued (13.8%) and stagnant (0%) growth of the urology workforce based on the American Urological Association Annual Census data in 2019 and the US Census Bureau’s projections showed that within the context of the impending urology workforce shortage, there will be an exaggerated shortage of total urologists per capita for populations aged 65 years and older.

**Meaning:**

These findings highlight the need for structural changes and advocacy to increase the available urology workforce.

## Teaser

This cross-sectional study uses 2 stock and flow models to project the size and demographic characteristics of the urology workforce per capita through 2060 and to anticipate the timing and degree of the impending urology workforce shortage.

## Introduction

Multiple estimates have found impending workforce shortages across surgical fields due to the Balanced Budget Act of 1997, which limits the funding necessary to train residents and caps the number of government-subsidized residency positions. This workforce shortage will be exacerbated by the silver tsunami, where by 2030, the youngest members of the Baby Boomer generation will be in the Medicare age group that heavily uses health care services.^1,2,3^ Urology is no exception, and our current supply of practicing urologists of 13 044 is still far short of the 14 400 urologists that the US Department of Health projected to be necessary to meet the demand for urological services.^1^ The American Urological Association (AUA) has recognized the workforce shortage as a federal advocacy priority, with only 38% of all US counties having practicing urologists, recent declines in the number of urologists per capita, and older median age of urologists.^4^ In addition, because the Medicare population uses urological services 3 times more often than the general population, there have been projections that even if we maintain the current number of urologists per capita, there will be a shortage of urologists by 46% in 2035.^5^ This has substantial downstream consequences on access to care, delays for surgical evaluation, longer travel time for rural patients, and heightened pressure on practicing urologists to meet the increased demands, placing them at risk for burnout.^6^ In addition to concerns of the workforce shortage, there have been concerns about how the future urology workforce can better reflect our patient population, particularly with regard to race and gender.^7^ Despite growth of female representation in urology compared with other specialties, it continues to be a heavily male-dominated field with only 9.9% of practicing urologists being female.^8,9^ Female urologists continue to be underrepresented relative to the 30% of urological patient population being female.^7^

There has not been an updated projection of the urology workforce per capita beyond 2035 with an understanding of the present-day urology workforce. The US Department of Health and Human Services recognizes that at least a decade is required to enact policies and programs to increase the physician workforce, given the length of training and time required to change physician training infrastructure.^1^ Therefore, it is time critical to have a nuanced understanding of the impending workforce shortage in urology. Our study projects the urology workforce per capita and demographic representation over the next 40 years under guided assumptions with 2 stock and flow models. We hypothesize that in our continued growth model, there will be a recovery beyond the current 2020 urology workforce per capita, whereas in our growth stagnant model, we will see a continued decline in the urology workforce per capita. We also hypothesize that the urology workforce shortage per capita will be more severe for the population aged 65 years and older. Finally, we hypothesize that in the context of a decreasing number of urologists per capita in the next 4 decades, there will be an overall growth per capita of female urologists.

## Methods

Because we used publicly available data, our institution deemed this analysis exempt from institutional review board oversight. Informed consent was waived by our institution for this reason. This study followed the Strengthening the Reporting of Observational Studies in Epidemiology (STROBE) reporting guidelines.

### Current Practicing Urologists and US Population Data

According to the 2019 AUA Census, which defines the urologist population by National Provider Identifier, valid medical licenses of both urologists and pediatric urologists, and American Board of Urology certification records, there are currently 13 044 practicing urologists, including 11 758 men (90.1%) and 1286 women (9.9%).^10^ The AUA Census provides the age distribution for all practicing urologists. These were used to estimate the number of practicing urologists by age and gender in 2020. Each gender and age are divided proportionally into 5-year age categories and used as the estimate of practicing urologists by gender and age group in 2020. For US population data, we used the US Census Bureau 2017 national population projections based on the US Census data from 2010 using a cohort-component method and assumptions about demographic components of change, such as future trends in births, deaths, and net international migration from 2017 to 2060.^11^

### Stock and Flow Model

The stock and flow model estimates the number of practicing urologists in a year with the addition of urology residents entering the current practicing urologist population and subtraction of the retiring urologists, as follows: urologists*_i_*_ + 1_ = urologists*_i_* + residents*_i_* − retirees*_i_*, where *i* = half-decade increment in time. Given that 311 urologists entered the workforce in 2018 according to the Accreditation Council for Graduate Medical Education Data Resource Book, we assume that 320 urologists would enter the workforce annually.^8,10,12^ Among those entering workforce in 2018, 24.4% were female.^12^ The median age of those entering the workforce is 32 years.^8^ We assume the future proportion will continue to be 25% female and 75% male and that they enter at 32 years of age.^8^ For the growth model, we assume continued growth of urologists entering the workforce by 13.8% every 5 years using the Accreditation Council for Graduate Medical Education growth rate from 2013 to 2018.^12^ For the stagnant model, the number of incoming urologists is constant at 320.

The flow portion of the model subtracts the retiring urologists from the population using the 2019 AUA Census. The 2019 AUA Census provided the proportion of urologists in 5-year increments of planned age at full retirement separately by gender. On the basis of the stable planned retirement age in AUA census from 2016 to 2020, we assume that these retirement proportions would remain constant throughout the time projected.^10,13,14^ We performed a sensitivity analysis to see how this projection would change with the planned retirement age of 70 years.

### Per Capita Estimates

The per capita estimates are calculated using the estimated urologist population and dividing by the US population. We calculated total urologists, male urologists, and female urologists per total capita. We also calculated the number of urologists per the population aged 65 years and older and urologists per matching gender per capita. We then calculated the number of urologists needed to maintain the current urologists per capita and how many additional urology residency slots would need to be added annually to maintain the current level of 4 urologists per 100 000.

### Statistical Analysis

Stock and flow models were generated in Excel software version 2016 (Microsoft). Data analysis was performed from June 2020 to March 2021.

## Results

In 2019, according to the AUA census, there were 3.99 urologists per 100 000 in the US (total, 13 044 urologists; 11 758 male [90.1%] and 1286 female [9.9%]).^8^ The median age range of urologists was 55 to 59 years. In 2020, using our assumptions listed in the Table, we project that there were 13 365 total practicing urologists, with 11 999 (89.8%) being men and 1366 (10.2%) being women.

**Table.** Key Forecast Assumptions

Variable	Key assumption	Sources
Baseline practicing urologists, No.	13 044	2019 AUA Census^10^
Male vs female practicing urologists, No. (%)		2019 AUA Census^10^
Male	11 758 (90.1)	
Female	1286 (9.9)	
Age of male vs female practicing urologists, median, y		2019 AUA Census^10^
Male	55-59	
Female	40-44	
Baseline new annual urologists	320 (vs 311 on ACGME 2018)	2018 ACGME workbook^8^
Age of male vs female new urologists annually, y	32	2019 AUA Census^10^
Graduating resident No. growth per 5 y, %	0 Baseline growth	
	13.8 Baseline growth	ACGME workbook; from 2013 to 2018, 13.8% growth in No. of active urology residents^8^
New female urologists annually, %	25 (vs 24.4 on ACGME 2018)	2018 ACGME workbook^8^
Age of male vs female planned age of retirement, median, y		2019 AUA Census^10^
Male	69	
Female	65	

Abbreviations: ACGME, Accreditation Council for Graduate Medical Education; AUA, American Urological Association Annual.

**Figure 1.**
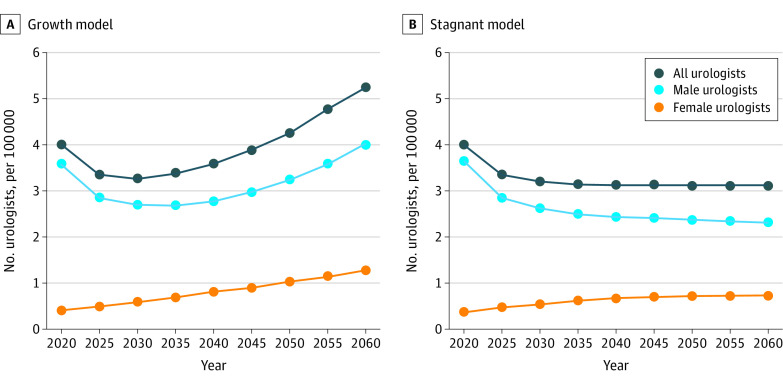
Projected Number of Urologists per Capita From 2020 to 2060 A, Graph shows projected number of urologists under the continued 13.8% growth stock and flow model. B, Graph shows projected number of urologists under the 0% stagnant growth stock and flow model. In both panels, each dot represents the number of urologists per 100 000 persons corresponding to the year.

**Figure 2.**
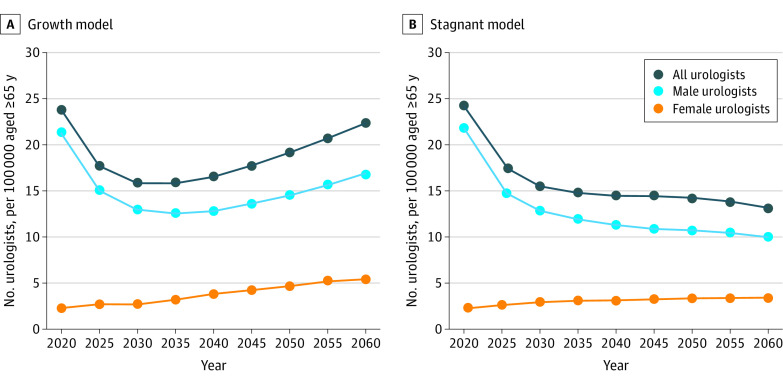
Projected Number of Urologists per 100 000 People Aged 65 Years or Older From 2020 to 2060 A, Graph shows projected number of urologists under the continued 13.8% growth stock and flow model. B, Graph shows projected number of urologists under the 0% stagnant growth stock and flow model. In both panels, each dot represents the number of urologists per 100 000 persons aged 65 years or older corresponding to the year.

**Figure 3.**
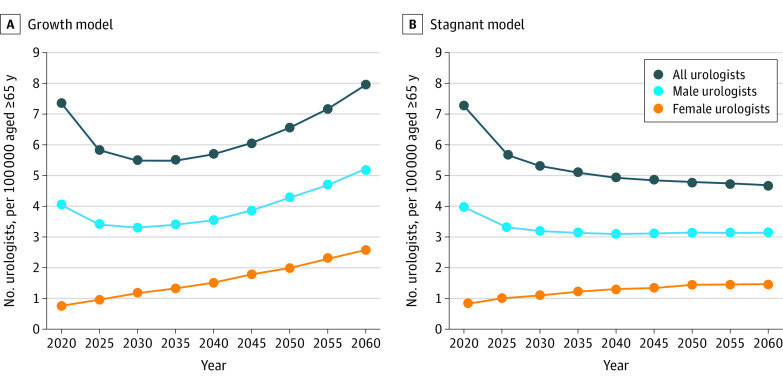
Projected Number of Urologists per Capita by Matching Gender From 2020 to 2060 A, Graph shows projected number of urologists under the continued 13.8% growth stock and flow model. B, Graph shows projected number of urologists under the 0% stagnant growth stock and flow model. In both panels, each dot represents the number of urologists per 100 000 persons by matching gender corresponding to the year.

In our continued growth model of 13.8% more urologists joining practice every 5 years, 2030 will have the lowest urologists per capita of 3.3 urologists per 100 000 persons (Figure 1A). By 2060, there will be 5.2 urologists per 100 000 persons. For the Medicare population, there are currently 23.8 urologists per 100 000 persons aged 65 years and older in 2020 (Figure 2A). This ratio will be the lowest in 2035, with 15.8 urologists per 100 000 persons aged 65 years and older, and increases to 22.3 by 2060 and never recovers to its 2020 baseline level. When matching female urologists to the female population, there are 0.8 female urologists for 100 000 female persons in 2020, which increases at each time interval to 2.5 female urologists to 100 000 female persons by 2060 (Figure 3A).

In our stagnant growth model of 0%, there will be a continued decrease of urologists per capita to 3.1 urologists per 100 000 persons in 2035 and beyond (Figure 1B). For the Medicare population, there is a continued decrease at each time point with 13.1 urologists per 100 000 persons aged 65 years and older by 2060 (Figure 2B). When matching female urologists to the female population, there is continued growth that plateaus at 1.5 female urologists to 100 000 female persons in 2050 and beyond (Figure 3B).

Our sensitivity analysis examining retirement age of 70 years showed no significant changes from our primary analyses and conclusions. Finally, we found that to maintain the current urologists per capita to 2060, an additional 3851 urologists are required. This translates to an increase of at least 96 urology residency slots annually until 2060.

## Discussion

To our knowledge, this cross-sectional study is the first to project the urology workforce per capita and demographic characteristics of urologists over the next 40 years and has 3 key findings. First, the total number of practicing urologists per capita will decrease in the coming decades, even with sustained growth of the resident complement across urology training programs, and will not recover to baseline until 2060. Second, there will be an exaggerated shortage of total urologists per population aged 65 years and older in both models of our projections. Finally, female urologists per capita will continue to increase in the context of decreasing urologists per capita in both continued growth and growth stagnant models. Collectively, these projections highlight the severity of the impending shortage of urologists and importance of structural change and advocacy to maximize our available urology workforce. On the basis of our model, we found that to maintain the current urologists per capita to 2060, an additional 3851 urologists are required. To meet the demands of more urologists, we would need to increase at least 96 urology residency slots annually.

Our analysis demonstrates that prior efforts to increase the urology workforce have been insufficient, with problems escalating in the decades to come. Specifically, despite an additional 14 accredited urology residency programs between 2013 and 2018, our current supply of practicing urologists of 13 044 is still far short of the 14 400 urologists projected necessary to meet the demand for urological services.^1,12^ Our projections demonstrate that the disparity will worsen in the coming decades even with continued growth of 13.8% graduating urologists every 5 years.^1^ Because of the number of retiring urologists, the number of urologists per capita will not reach baseline 2020 levels until 2050. Without additional growth of training positions, the workforce shortage will become even more severe, with a continued decline in urologists per capita through 2060. We provided these 2 alternate models for workforce projections, understanding that the actual urology workforce will most likely fall between these 2 projections. Regardless, our field must be prepared to face a growing shortage of physicians for the next 40 years, and possibly beyond.

The impending shortage will be felt most keenly by the elderly and most medically vulnerable patients. Given the increased prevalence of urological conditions in an aging population, the Medicare population heavily uses urology services.^15^ Etzioni et al^16^ found that the group of patients aged 65 years and older used 64.8% of all urological services. McKibben et al^5^ reaffirmed that adults aged 65 years and older use urological services at a rate 3 times higher than the general population. This is consistent with findings of Urologic Diseases in America project,^5^ which documented substantial and growing incidence and prevalence of a number of urological conditions, such as benign prostatic hyperplasia, incontinence, and urological cancers, that affect the older population. Although it is possible that some Medicare physicians overuse urological services, this finding has been confirmed by multiple authors and is consistent with how commonly urological conditions affect elderly patients more than the rest of the population. Both of our models show a decline in urologists per 100 000 persons aged 65 years and older in the coming years, which is particularly concerning given that this population heavily uses urology services, thereby exacerbating the existing shortage of urologist supply relative to the demand.^5^ This has major downstream consequences on access to care, delays for surgical evaluation, and potential for worse patient outcomes.^6^

It is worthwhile to consider the impact of telemedicine in this context. Although urology has pioneered the integration of telemedicine to provide care for our patients, it is still largely unknown whether video visits in urology can serve as a substitute for clinic evaluations and how it affects clinical efficiency.^17,18^ In addition, the patients who use telemedicine to access care tend to be younger and female, which may still preclude providing care for the elderly patient population.^17^ However, we anticipate that telemedicine will continue to be an integral part of providing care for our patients and will be further used by the Medicare population as this population becomes more accustomed to technological advances required for telemedicine.

One positive aspect of the projections in this study is that the number of female urologists consistently increases in both projection models. Although both male and female urologists provide urological care for diverse patient populations, there are substantial differences in practice patterns by gender. Almost one-half of female urologists see a majority of female patients as part of their practice, whereas only 3.5% of male urologists see a majority of female patients as part of their practice.^10^ Although this is partially owing to more female urologists subspecializing in female pelvic medicine and reconstructive surgery, when comparing general urologists of each gender, female general urologists logged 2.2 times the number of urogynecological cases compared with their male counterparts.^19,20^ Currently, with 0.8 female urologists per 100 000 female population, there is substantial underrepresentation of female urologists for a gender-concordant population in the US.^7^ This is particularly noteworthy given that 30% of urology patients are female, and patient surveys have highlighted patient preference for gender-concordant urologists for urinary incontinence.^21^ With an increasing number of female urologists in our projection, they have not only increased availability to provide care for female patients but also increase the likelihood of mutually respective care for diverse patient populations by contributing to the diversity of the urology workforce.^9^

### Limitations and Strengths

Our study has several limitations. The projections of the workforce model are dependent on assumptions listed in the Table. Although 24.4% of the current urology resident workforce is female, which is much higher than previously, we did not think that this growth in representation would be linear. Therefore, we assumed that approximately 25% of the resident workforce will be female, understanding that this could be an underestimate. We also used planned retirement age as a surrogate for actual retirement age because that was the closest data we had available. We assumed that because the planned retirement age was stable from 2016 to 2020, it would remain constant throughout the projection.^13,14^ If all urologists delay retiring until age 70 years, the greatest increase made would be, at most, 2% more urologists in 2060 compared with our original models. Thus, the results and conclusions do not change overall. There are limited longitudinal data for some of our assumptions, but the 2 scenarios of continued and stagnant growth were modeled to account for possible variability, understanding that the actual urology workforce will most likely fall between these 2 models. Next, we cannot account for the changes in urologist practice variation with the increasing number of practicing urologists. For example, approximately 10% of the urologists who currently plan to retire at age 70 or beyond listed that their reasoning for continuing to practice is their inability to recruit a replacement.^10^ If there are more urologists available, the decision-making process regarding retirement or total work hours could be affected, which is not accounted for in our modeling. Finally, our US population projection is based on the US Census Bureau projections from 2017, which could deviate from the actual population in the future but is the best estimate and projection of each time point available.

These limitations notwithstanding, our findings highlight the time sensitivity and importance of continued advocacy for increased graduate medical education funding and other policies to ensure that we effectively mitigate the impending urology workforce shortage by funding additional urology residency positions. Without such interventions, there will be negative downstream consequences for patient care and outcomes.^6^ One clinical example is evaluation of hematuria, a common diagnosis that leads to referral from primary care or the emergency department and requires further urological workup to identify 1 in 10 patients who may have a life-threatening malignancy or other treatable condition.^22^ Many studies looking at diagnostic evaluation, such as cystoscopy, of patients with hematuria, have already identified multifactorial reasons for delay in full evaluation that have led to later stage of diagnosis, higher disease burden, and less favorable cancer outcomes.^22,23,24,25^ Given the impending urology workforce shortage, we can project that the further delay in patient care would lead to worse patient outcomes. Continued utilization of advanced practice practitioners may bridge the gap between patient’s access to care by serving as vital partners in providing quality care to patients.^5^ However, it is crucial to sustain growth in the number of urologists alongside that of advanced practice practitioners given that urologists are key practitioners of surgical services and the extent of advanced practice practitioners’ practice within urology is understudied at this time.^26^ Physician burnout remains a constant threat to a stable urology workforce.^14^ Creating organizational cultures where urologists are supported through greater autonomy and flexibility, improvements in work-life balance, more diverse and inclusive work communities, and greater efficiency will help buffer against burnout and lead to a more robust, stable, and productive workforce.

## Conclusions

In our projection of the urology workforce to 2060, we found that the total number of practicing urologists per capita will decrease in the coming decades, with a nadir in the year 2030, even with sustained growth of the resident complement across urology training programs. Second, there will be an exaggerated shortage of total urologists for the population aged 65 years and older in both models of our projections. Finally, the number of female urologists per female capita will continue to increase in the context of decreasing urologists per capita in both continued growth and stagnant growth models. Given the length of training and time required to change physician training and practice infrastructure, there is an urgent need for advocacy for increasing the graduate medical education budget to train more urologists and mitigate other factors, such as burnout, that contribute to the urology workforce shortage.

## References

[1] US Department of Health and Human Services; Health Resources and Services Administration; Bureau of Health Professions. Physician supply and demand: projections to 2020. October 2006. Accessed October 18, 2021. https://bhw.hrsa.gov/sites/default/files/bureau-health-workforce/data-research/physician-2020-projections.pdf

[2] Williams TE Jr, Satiani B, Thomas A, Ellison EC. The impending shortage and the estimated cost of training the future surgical workforce. Ann Surg. 2009;250(4):590-597. doi:10.1097/SLA.0b013e3181b6c90b 19730238

[3] Vespa J. The graying of America: more older adults than kids by 2035. March 13, 2018. Accessed June 29, 2020. https://www.census.gov/library/stories/2018/03/graying-america.html

[4] American Urological Association. Our priority: address the urologic workforce shortage. Accessed December 8, 2020. https://www.auanet.org/advocacy/federal-advocacy/workforce-shortages

[5] McKibben MJ, Kirby EW, Langston J, . Projecting the urology workforce over the next 20 years. Urology. 2016;98:21-26. doi:10.1016/j.urology.2016.07.028 27491965

[6] Mahoney ST, Strassle PE, Schroen AT, . Survey of the US surgeon workforce: practice characteristics, job satisfaction, and reasons for leaving surgery. J Am Coll Surg. 2020;230(3):283.e1-293.e1. doi:10.1016/j.jamcollsurg.2019.12.003 31931143

[7] Breyer BN, Butler C, Fang R, . Promotion disparities in academic urology. Urology. 2020;138:16-23. doi:10.1016/j.urology.2019.10.042 31917291

[8] American Urological Association. 2019: The state of urology workforce and practice in the United States. April 14, 2020. Accessed October 18, 2021. https://auanet.org/research/research-resources/aua-census/census-results

[9] Halpern JA, Lee UJ, Wolff EM, . Women in urology residency, 1978-2013: a critical look at gender representation in our specialty. Urology. 2016;92:20-25. doi:10.1016/j.urology.2015.12.092 26952568

[10] American Urological Association. 2018: The state of urology workforce and practice in the United States. April 5, 2019. Accessed October 18, 2021. https://auanet.org/research/research-resources/aua-census/census-results

[11] US Census Bureau. 2017 National population projections: projected population by single year of age, sex, race, and Hispanic origin for the United States—2016 to 2060. Revised October 8, 2021. Accessed October 18, 2021. https://www.census.gov/data/datasets/2017/demo/popproj/2017-popproj.html

[12] Accreditation Council for Graduate Medical Education. ACGME data resource book: academic year 2017-2018. 2018. Accessed October 18, 2021. https://www.acgme.org/globalassets/pfassets/publicationsbooks/2017-2018_acgme_databook_document.pdf

[13] American Urological Association. 2020: The state of urology workforce and practice in the United States. May 27, 2021. Accessed October 18, 2021. https://auanet.org/research/research-resources/aua-census/census-results

[14] American Urological Association. 2016: State of the urology workforce and practice in the United States. April 11, 2017. Accessed October 18, 2021. https://auanet.org/research/research-resources/aua-census/census-results

[15] Pruthi RS, Neuwahl S, Nielsen ME, Fraher E. Recent trends in the urology workforce in the United States. Urology. 2013;82(5):987-993. doi:10.1016/j.urology.2013.04.080 24055244

[16] Etzioni DA, Liu JH, Maggard MA, Ko CY. The aging population and its impact on the surgery workforce. Ann Surg. 2003;238(2):170-177. doi:10.1097/01.SLA.0000081085.98792.3d 12894008 PMC1422682

[17] Andino JJ, Lingaya MA, Daignault-Newton S, Shah PK, Ellimoottil C. Video visits as a substitute for urological clinic visits. Urology. 2020;144:46-51. doi:10.1016/j.urology.2020.05.080 32619595 PMC7834609

[18] Andino JJ, Castaneda PR, Shah PK, Ellimoottil C. The impact of video visits on measures of clinical efficiency and reimbursement. Urol Pract. 2021;8(1):53-57. doi:10.1097/UPJ.0000000000000149 33363249 PMC7757337

[19] Liu JS, Dickmeyer LJ, Nettey O, Hofer MD, Flury SC, Kielb SJ. Disparities in female urologic case distribution with new subspecialty certification and surgeon gender. Neurourol Urodyn. 2017;36(2):399-403. doi:10.1002/nau.22942 26678743

[20] Rotker K, Iosifescu S, Baird G, Thavaseelan S, Hwang K. What’s gender got to do with it: difference in the proportion of traditionally female cases performed by general urologists of each gender. Urology. 2018;116:35-40. doi:10.1016/j.urology.2017.12.040 29550347

[21] Ficko Z, Li Z, Hyams ES. Urology is a sensitive area: assessing patient preferences for male or female urologists. Urol Pract. 2018;5:139-142. doi:10.1016/j.urpr.2017.02.005 37300200

[22] Friedlander DF, Resnick MJ, You C, . Variation in the intensity of hematuria evaluation: a target for primary care quality improvement. Am J Med. 2014;127(7):633-640. doi:10.1016/j.amjmed.2014.01.010 24486290 PMC4074456

[23] Cole E, Hopman W, Kawakami J. High resolution analysis of wait times and factors affecting surgical expediency. Can Urol Assoc J. 2011;5(1):13-17. doi:10.5489/cuaj.553 21470506 PMC3036749

[24] David SA, Patil D, Alemozaffar M, Issa MM, Master VA, Filson CP. Urologist use of cystoscopy for patients presenting with hematuria in the United States. Urology. 2017;100:20-26. doi:10.1016/j.urology.2016.09.018 27645524

[25] Santos F, Dragomir A, Kassouf W, Franco E, Aprikian A. Urologist referral delay and its impact on survival after radical cystectomy for bladder cancer. Curr Oncol. 2015;22(1):e20-e26. doi:10.3747/co.22.2052 25684993 PMC4324349

[26] Hollenbeck BK, Kaufman SR, Oerline M, . Effects of advanced practice providers on single specialty surgical practice. Ann Surg. Published online March 3, 2021. doi:10.1097/SLA.0000000000004846 33914476 PMC8989058

